# Vitamin D and LL-37 in Serum and Saliva: Insights into Oral Immunity

**DOI:** 10.3390/cimb47020102

**Published:** 2025-02-06

**Authors:** Sotiria Davidopoulou, Kali Makedou, Areti Kourti, Ioanna Gkeka, Panagiotis Karakostas, Maria Pikilidou, Kosmas Tolidis, Sotirios Kalfas

**Affiliations:** 1 Department of Operative Dentistry, School of Dentistry, Aristotle University of Thessaloniki, 541 24 Thessaloniki, Greece; ktolidis@dent.auth.gr; 2 Laboratory of Biochemistry, School of Medicine, Aristotle University of Thessaloniki, 541 24 Thessaloniki, Greece; kmakedou@auth.gr (K.M.); aretikourti@auth.gr (A.K.); gkekagi@gmail.com (I.G.); 3 Department of Preventive Dentistry, Periodontology and Implant Biology, School of Dentistry, Aristotle University of Thessaloniki, 541 24 Thessaloniki, Greece; karako.dent@gmail.com (P.K.); kalfas@dent.auth.gr (S.K.); 4 Hypertension Excellence Centre, Second Department of Nephrology, AHEPA Hospital, Aristotle University of Thessaloniki, 541 24 Thessaloniki, Greece; pikilidou@gmail.com

**Keywords:** 25-hydroxyvitamin D, LL-37 antimicrobial peptide, saliva, serum

## Abstract

(1) Background: In recent years, there has been a growing interest in understanding the innate immunity of the mouth, particularly the mechanisms through which vitamin D influences oral health. Researchers have increasingly focused on the association between vitamin D and the antimicrobial peptide LL-37 since the CAMP gene, responsible for encoding the LL-37 peptide, is a direct target of both vitamin D and its receptor (vitamin D receptor, VDR). This study aimed to explore the correlation between the 25-hydroxyvitamin D (25(OH)D) levels and the concentration of the LL-37 peptide in both serum and saliva. The objective was to compare the serum concentrations of 25(OH)D and ll-37 with those in saliva and to access the correlations between the two compounds. (2) Methods: Serum and whole saliva samples were collected from 72 healthy adults (mean age 28.68 ± 8.35). The levels of 25(OH)D and LL-37 were assessed in both the saliva and serum samples using commercially available enzyme-linked immunosorbent assay (ELISA) kits. (3) Results: The 25(OH)D levels in the serum (median 5.92 ng/mL, min–max 2.7–10.4 ng/mL) correlated with the LL-37 serum levels (62 ng/mL, min–max 18–378 ng/mL; Pearson’s r 0.328, *p* = 0.005). Additionally, the 25(OH)D levels in saliva (median 1.16 ng/mL, min–max 0.54–2.12 ng/mL) strongly correlated with the LL-37 salivary levels (median 44 ng/mL, min–max 6.5–205 ng/mL; Pearson’s r 0.667, *p* < 0.001). The 25(OH)D salivary levels demonstrated a robust correlation with the LL-37 salivary levels. (4) Conclusions: This discovery emphasizes the complex interplay between vitamin D and LL-37 and lay the groundwork for the further exploration of vitamin D’s role in oral immune function.

## 1. Introduction

Antimicrobial peptides (AMPs), also referred to as host defense peptides, are essential components of the humoral branch of innate immunity. They possess the remarkable capability to rapidly eliminate or neutralize microorganisms while simultaneously regulating immune responses [1]. Several prominent families of AMPs have been identified in humans including defensins, histatins, cathelicidins, hepsidins, and others [2]. In humans, LL-37 is the sole cathelicidin discovered to date [3]. This peptide is synthesized by a variety of tissues and cells, such as epithelial cells lining the skin, gastrointestinal tract, respiratory system, oral cavity, and urogenital tract, as well as by various innate immune cells. LL-37 is a multifunctional component of innate immunity, capable of acting as a potent pro-inflammatory agent or as an anti-inflammatory regulator, depending on the surrounding microenvironment [1,4,5]. Furthermore, LL-37 plays a pivotal role in bridging innate and adaptive immunity and is also involved in facilitating wound healing. The diverse functions of LL-37 are intricately linked to its regulatory mechanisms, which vary according to specific microenvironmental conditions such as the oral environment [6]. Due to its critical role in maintaining immune homeostasis, the dysregulation of LL-37 activity has been implicated in the onset and progression of numerous human diseases, such as the severe periodontal disease observed in Kostmann disease patients and Papillon-Lefevre syndrome, who lack or have very low levels of LL-37 [7,8,9]. LL-37 plays an undisputable role in maintaining oral health and combating oral diseases and has been proposed as a marker for inflammation severity and treatment outcomes in oral diseases [6]. Recent research suggests a complex relationship between LL-37 and vitamin D [10,11,12,13] as 1,25-dihydroxyvitamin D3 is a direct inducer of antimicrobial peptide gene expression [13].

The role of vitamin D in overall health, calcium homeostasis, and maintaining bone integrity has been well-recognized for decades [10]. Researchers have highlighted the importance of this steroid hormone in supporting the proper functioning of various organ systems. In addition to reducing inflammation and managing pathogens, vitamin D plays a crucial role in enhancing the body’s ability to combat diseases through immune response pathways. Evidence suggests that vitamin D-dependent immune responses can be activated in the presence of local infections [11]. Moreover, it has been demonstrated that active vitamin D (1,25(OH)_2_D_3_) influences the induction of the only human cathelicidin LL-37 and inflammatory cytokines in response to local infections [11]. In humans, active vitamin D is produced from circulating, inactive vitamin D, 25-hydroxyvitamin D (25(OH)D) by 1-hydroxylase (Cyp27B1) [12]. This suggests a novel mechanism where the local conversion of inactive vitamin D to its active form modulates immune function at the site of infection [11]. In 2004, Wang and colleagues identified consensus vitamin D response elements (VDREs) within the cathelicidin LL-37 antimicrobial peptide (CAMP) gene that encodes the human cationic antimicrobial protein (hCAP-18) with a molecular weight of 18 kDa [13]. Their research also demonstrated that vitamin D plays a direct role in enhancing CAMP protein expression in monocytes, neutrophils, keratinocytes, and squamous carcinoma cells [13]. hCAP-18 undergoes proteolytic processing, leading to the release of the antimicrobial peptide LL-37. The active form of vitamin D (1,25(OH)_2_D_3_), has been shown to act as a strong inducer of LL-37 production in human bronchial epithelial cells, myeloid cells, and colon cancer cell lines [13,14]. Epidemiological studies in healthy populations have consistently highlighted a correlation between vitamin D deficiency and reduced serum LL-37 levels [15,16]. Furthermore, a clinical trial involving septic patients demonstrated that vitamin D supplementation led to a significant increase in LL-37 levels compared with a placebo [17]. These findings underscore the critical role of nutrition in regulating LL-37.

Recent studies have explored the potential of salivary vitamin D measurements as a non-invasive alternative to serum tests. While vitamin D is measurable in saliva, its reliability as a biomarker remains uncertain. Some researchers have found no significant correlation between the salivary and serum vitamin D levels while others reported a significant positive correlation [18,19]. A moderate correlation between the salivary and serum 25(OH)D levels was observed in healthy individuals, suggesting that salivary 25(OH)D assays could potentially replace serum tests in this population [20]. Despite these findings, further research is needed to establish the reliability and clinical utility of salivary vitamin D measurements across various health conditions.

Salivary levels of LL-37, an antimicrobial peptide, have emerged as a promising biomarker for assessing oral health conditions [6]. Elevated LL-37 levels were demonstrated in patients with periodontitis [21,22] and oral lichen planus [23]. Conversely, smoking has been shown to adversely affect the salivary LL-37 levels [21]. In children, the salivary LL-37 levels positively correlate with age and appear to provide a protective effect against dental caries [24]. Collectively, these findings underscore the value of salivary LL-37 as a potential diagnostic tool for oral health evaluation and reinforce the significance of LL-37 in reflecting the oral immune response [6].

The rationale for this study stems from the pivotal role that vitamin D and the antimicrobial peptide LL-37 plays in modulating immune responses, particularly in the oral cavity. Vitamin D has been shown to regulate the expression of the CAMP gene, which encodes LL-37, but while vitamin D deficiency is widespread globally, its potential impact on the LL-37 concentrations in both systemic circulation and saliva has not been fully elucidated. Given that saliva serves as a first-line defense mechanism in maintaining oral health and is a mirror of the oral immune response, understanding how vitamin D influences the LL-37 levels locally in the oral cavity could provide critical insights into the interaction between nutritional status and innate immunity. This study was designed to investigate the correlation between the 25(OH)D and LL-37 levels in serum and saliva, exploring the potential impact of vitamin D deficiency on LL-37 concentrations in systemic circulation and in the oral cavity, providing insights into the role of vitamin D in immune modulation and oral health.

## 2. Materials and Methods

### 2.1. Study Population

**Figure 1:**
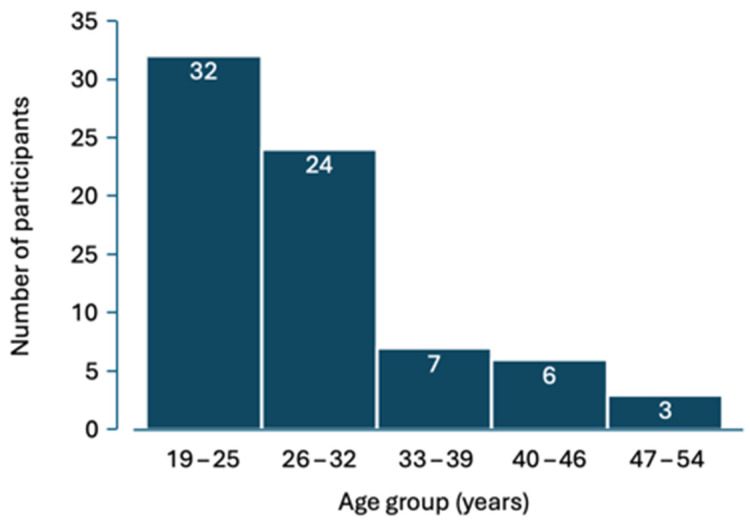
Distribution of the participants based on their age.

This study included 72 healthy adults with a mean age of 28.68 ± 8.35 years (28 males and 44 females), comprising students and personnel from the Dental School of Aristotle University of Thessaloniki. The distribution of the participants by their age is shown in Figure 1.

Sampling was conducted at the facilities of the dental school over two consecutive days, 12–13 March, in the morning, around 10:00 to 11:00 a.m., to minimize potential variations in peptide concentrations caused by circadian fluctuations [25]. All participants were in good general health, with no history of systemic diseases, and had not received any medications. Subjects who reported antibiotic intake or vitamin D supplementation within the previous three months as well as pregnant or lactating women were excluded from the study.

The study was conducted in accordance with the Declaration of Helsinki and approved by the Committee on Research Ethics of Aristotle University of Thessaloniki in Greece (protocol code 260634/2021, 3 November 2021). Informed consent was obtained from all participants before enrollment in the study.

### 2.2. Determination of Serum and Salivary 25(OH)D and LL-37

Blood samples were collected in tubes without anticoagulant after an 8-h fast and were centrifuged for 10 min in 4000 rpm. Serum was isolated and stored in −75 °C until analyzed. On the day of analysis, the samples were thawed and proceeded to ELISA analysis.

Unstimulated whole saliva samples were collected from each participant using the spitting method [24]. The collected samples were immediately frozen at −75 °C. Prior to analysis, the samples were thawed and centrifuged at 14,000× *g* for 5 min. Supernatants were then used to determine the concentration of both LL-37 and 25(OH)D.

The concentration of free LL-37 was measured using an enzyme-linked immunosorbent assay (ELISA) with commercially available kits (HyCult Biotechnology, Uden, the Netherlands), following the protocol provided by the manufacturer. Saliva samples, diluted fivefold, and serum samples, diluted 20 times, were tested alongside standards with concentrations ranging from 0.1 to 100 ng/mL. The concentration of 25(OH)D was also measured using an enzyme-linked immunosorbent assay (ELISA) using the IDK 25OHD immunoassay kit (Immundiagnostik, Bensheim, Germany). Saliva and serum samples, either undiluted or diluted fivefold, were tested alongside standards with concentrations ranging from 5 to 150 ng/mL. All tests were conducted in duplicate, and the average values were calculated. If the individual absorbance values deviated by more than 15% from the corresponding mean, the sample was retested.

### 2.3. Statistical Analysis

Data were summarized by means of descriptive statistical indices (median values, range and mean values, and standard deviation). Statistical comparisons between the salivary and serum levels of 25(OH)D and LL-37 were performed with the *t*-test. Correlations between the serum and salivary levels of 25(OH)D and LL-37 were examined using a 2-tailed Pearson’s (r) correlation coefficient. In all of the hypotheses testing procedures, the observed significance levels (*p*-value) were computed by the Monte Carlo simulation method. This approach leads to valid inferences even in cases where methodological presuppositions of the nonparametric tests are not satisfied [26]. The significance level of all statistical tests was predetermined at a = 0.05 (*p* ≤ 0.05). All data were processed with IBM SPSS Statistics software (version 28.0, Armonk, NY, USA: IBM Corp).

## 3. Results

**Figure 2:**
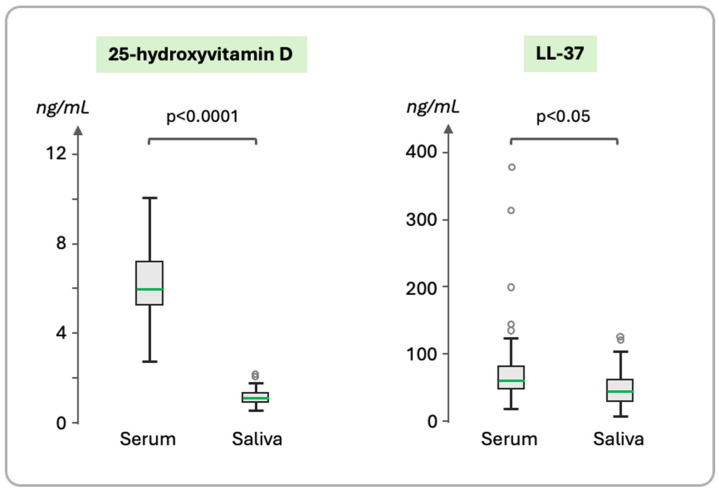
Concentrations of 25(OH)D and LL-37 in serum and saliva. The median values (green line), quartiles Q1 and Q3 (boxes), and whiskers (bars) indicating the variability outside the upper and lower quartiles and outliers (circles) are shown. The level of significance *p* for the difference between the serum and saliva concentrations is indicated.

The serum and salivary concentrations of 25(OH)D and LL-37 are presented in Table 1. All participants were vitamin D deficient. LL-37 was detected in all of the saliva and serum samples, with its concentrations showing considerable variation (Figure 2). The concentration of both compounds was higher in the serum than saliva, the difference being highly significant for 25(OH)D (Figure 2).

**Figure 3:**
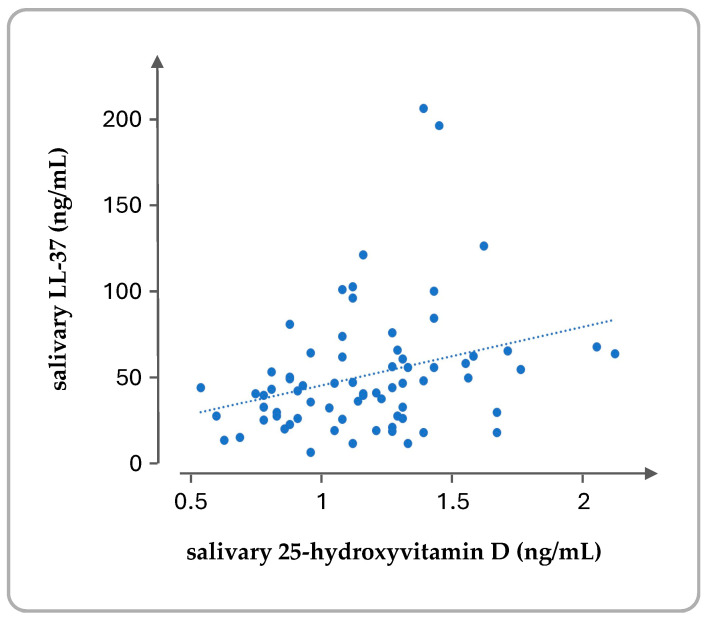
Correlation between the concentration (in ng/mL) of 25(OH)D and LL-37 in the saliva samples.

Significant correlations were also found between the serum levels of 25(OH)D and LL-37 (Pearson’s r = 0.328, *p* = 0.005) and between the salivary levels of 25(OH)D and LL-37 (Pearson’s r = 0.667, *p* < 0.001) (Figure 3). The correlation between the salivary levels was stronger than the corresponding ones in the serum. On the contrary, the serum 25(OH)D levels did not correlate with the salivary 25(OH)D levels (Pearson’s r = 0.175, *p* = 0.141). A weak correlation was also observed between the LL-37 concentration in serum and saliva (Pearson’s r = 0.373, *p* = 0.01).

No correlation was observed between the age or the gender of the participants and the concentrations of the compounds either in the serum or saliva.

## 4. Discussion

This study examined the relationship between the 25-hydroxyvitamin D (25(OH)D) and LL-37 levels in both serum and saliva to explore their potential interactions and implications in immune modulation and oral health. The LL-37 salivary and serum levels were found to considerably vary, consistent with all previous studies on LL-37 concentrations in serum and saliva [24,27,28,29,30,31]. One notable finding was that all of the participants demonstrated low vitamin D serum levels (<10 ng/mL). The vitamin D status of the participants was classified according to the recommendations of the Endocrine Society, with sufficiency defined as 30–100 ng/mL, insufficiency as 20–29 ng/mL, and deficiency as <20 ng/mL [32]. This deficiency may be attributed to the timing of the study, which was conducted in March, shortly after the winter season when reduced sunlight exposure often leads to diminished vitamin D synthesis. Research consistently shows a strong seasonal variation in vitamin D levels, with the lowest concentrations typically observed in late winter and early spring [33,34]. Additionally, subjects who had received vitamin D supplementation were excluded from the study to avoid confounding factors related to supplementation.

The most interesting finding of the present study was that the salivary 25(OH)D levels demonstrated a strong correlation with the salivary LL-37 levels. It was also noticed that the serum 25(OH)D levels correlated with the serum LL-37 concentrations. Research on the relationship between vitamin D and LL-37 levels has yielded mixed results. While some studies found a positive correlation between circulating 25(OH)D and LL-37 levels in healthy adults [16,29], others reported no significant association [35,36]. Notably, LL-37 levels were found to be lower in HIV-positive individuals not receiving antiretroviral therapy compared with healthy controls and HIV-positive individuals on treatment, while a direct relationship between circulating 1,25-dihydroxyvitamin D, 25(OH)D, and LL-37 was noted in both the HIV and healthy-control groups [29]. The study by Larcombe et al. on a Canadian First Nation population revealed that certain polymorphisms in the vitamin D receptor and vitamin D binding protein genes may influence the serum LL-37 levels and how these levels are altered after vitamin D supplementation [35]. In our study, the circulating 25(OH)D levels did not correlate with the salivary LL-37 levels. Our findings align with the study by Alhelfi et al. [27], which reported that while the salivary LL-37 levels were positively correlated with the serum vitamin D levels in females with vitamin D concentrations of 30 ng/mL or more, no such correlation was observed when the circulating vitamin D levels were below 10 ng/mL. To our knowledge, this is the first report to specifically address the correlation between the vitamin D and LL-37 levels in saliva. Although a correlation between local and systemic vitamin D levels was observed, the underlying mechanisms remain unclear and cannot be fully explained yet. However, it is well-established that the active form of vitamin D, 1,25(OH)_2_D, plays a crucial role in inducing the production of the antimicrobial peptide LL-37 across various cell types. In human peripheral blood mononuclear cells, supplementation with 25(OH)D has been shown to increase the intracellular expression of CAMP (the gene encoding LL-37) and hCAP18 (the precursor to LL-37), with the extracellular secretion of LL-37 further enhanced under bacterial or viral stimulation [37]. Similarly, in keratinocytes derived from diabetic foot ulcers, treatment with 1,25(OH)_2_D led to increased CAMP expression and LL-37 production, thereby promoting wound healing and antimicrobial activity [38]. In gingival epithelial cells, 1,25(OH)_2_D stimulation resulted in increased LL-37 mRNA expression and improved antibacterial activity against periodontal pathogens [39]. Moreover, in patients with severe congenital neutropenia, 1,25(OH)_2_D was found to induce pro-LL-37 expression in neutrophil precursors and transformed B cells, highlighting its potential as a therapeutic strategy [40].

Recent studies have shown promising correlations between the salivary and serum levels of various biomarkers, suggesting saliva as a potential non-invasive alternative to blood tests [41]. According to our results, the serum 25(OH)D levels did not correlate with the salivary 25(OH)D levels. This observation is consistent with controversial findings in the literature regarding vitamin D levels in saliva and serum. Recent studies have investigated the correlation between serum and salivary vitamin D levels, yielding mixed results. While some research found no significant relationship between the salivary and serum vitamin D levels in healthy individuals and patients with hypertension [18], other studies reported a moderate correlation between the salivary and serum 25(OH)D levels in healthy people with low vitamin D intake [42,43]. These findings suggest that salivary 25(OH)D assays could potentially serve as a non-invasive alternative to serum testing in healthy individuals. However, the correlation between salivary and serum 1,25-dihydroxyvitamin D levels was found to be weak [42]. In patients with recurrent aphthous stomatitis, a significant positive correlation between the serum and salivary vitamin D levels was observed, although the salivary levels did not differ significantly between the patients and healthy controls [19]. These studies highlight the potential of salivary vitamin D testing but also underscore the need for further research to establish its reliability across different populations and health conditions. On the other hand, our results showed that the serum LL-37 significantly correlated with the salivary LL-37 levels. This discrepancy between 25(OH)D and LL-37 underscores the complexity of vitamin D metabolism and its differing localized versus systemic roles in immunity. To date, no prior studies have directly examined the correlation between the LL-37 levels in saliva and serum, highlighting the need for further research in this area. While the LL-37 levels in both serum and saliva may serve as indicators of systemic immune activity, the distinct regulatory mechanisms influencing the 25(OH)D levels in saliva warrant further investigation.

Recent research suggests a bidirectional relationship between vitamin D and the gut microbiome. Vitamin D supplementation can modulate the upper gastrointestinal tract microbiome, decreasing the abundance of certain bacteria and increasing bacterial richness [44]. Vitamin D deficiency influences the microbiome composition and integrity of the gut epithelial barrier, primarily through the vitamin D receptor (VDR) [45]. The vitamin D–VDR axis regulates intestinal barrier function and controls innate and adaptive immunity in the gut [46]. Conversely, gut microbiota metabolites may regulate VDR expression [46]. Emerging research highlights the intricate interplay between vitamin D and the microbiome, raising the possibility of a similar interaction within the oral microbiome. Studies have shown that vitamin D supplementation can modify the gut microbiome by promoting the growth of beneficial bacteria [47]. It is conceivable that the oral microbiome may play a role in promoting the conversion of vitamin D into its active form, 1,25-dihydroxyvitamin D, or that vitamin D itself could influence the composition and activity of the oral microbiome. This interplay may significantly contribute to the regulation of oral immune responses and the maintenance of oral health. While serum 25(OH)D is the most widely used clinical marker for assessing overall vitamin D status, it only reflects the body’s vitamin D reserves and not the bioactive form. Since it is the active form of vitamin D, 1,25-dihydroxyvitamin D, that binds to the vitamin D receptor (VDR) and regulates gene expression, relying solely on the 25(OH)D levels may provide an incomplete picture. Further research is crucial to elucidate the interactions between the oral microbiome, the VDR, and vitamin D metabolism as well as their implications for both oral and systemic health.

In conclusion, this study highlights the complex interplay between vitamin D, immune modulation, and oral health. Our findings demonstrate a significant correlation between the salivary 25(OH)D and LL-37 levels and while exploratory, contribute to the broader understanding of how vitamin D influences immune function and offer valuable insights into the underlying mechanisms of vitamin D’s role in immune modulation in the oral environment. Furthermore, saliva may serve as a non-invasive medium for assessing certain immune markers. However, the lack of correlation between serum 25(OH)D and its salivary counterpart underscores the distinct regulatory mechanisms governing vitamin D metabolism in systemic versus localized environments. The observed vitamin D deficiency among participants further emphasizes the need to address the seasonal and lifestyle factors influencing vitamin D status. Moreover, the potential interaction between vitamin D and the oral microbiome presents a promising avenue for future research, as it may reveal novel insights into the mechanisms driving oral and systemic health. Collectively, these findings pave the way for the further exploration of vitamin D’s role in immune function and its broader implications for health and disease prevention.

## Figures and Tables

**Table 1:** Concentrations (in ng/mL) of 25(OH)D and LL-37 in serum and saliva. The concentrations are given as the mean ± standard deviation.

	25(OH)D (in ng/mL)	LL-37 (in ng/mL)
**Serum**	6.19 ± 1.58	74.17 ± 55.57
**Saliva**	1.17 ± 0.32	50.96 ± 36.31

## Data Availability

The data presented in this study are available on request from the corresponding author.
